# Biofluid Mechanics and Pathology of Venous Compression Syndromes: A Scoping Review

**DOI:** 10.7759/cureus.84402

**Published:** 2025-05-19

**Authors:** Kevin Rivera, Sam S Ahn

**Affiliations:** 1 Department of Emergency Medicine, The Ohio State University Wexner Medical Center, Columbus, USA; 2 Department of Surgery, Texas Christian University, Fort Worth, USA

**Keywords:** biomechanical force, biophysics, cellular pathology, cellular stress, chronic venous disease, general and vascular surgery, may-thurner syndrome, peripheral vascular surgery, vascular surgery, venous compression syndromes

## Abstract

Chronic venous disease (CVD) affects over 30 million individuals in the United States and imposes an annual economic burden of approximately $5 billion. Venous compression syndromes, defined as disorders caused by the extrinsic compression of veins at anatomically constrained sites, represent a significant yet underdiagnosed subset of CVD. Among these, May-Thurner Syndrome (MTS), the compression of the left common iliac vein by the right common iliac artery, is among the most clinically recognized. These syndromes often evade early detection due to the lack of specific non-invasive findings, resulting in delays between onset and diagnosis.

This scoping review integrates current knowledge on the biophysical, molecular, and cellular mechanisms involved in venous compression syndromes, with a focus on how altered biofluid mechanics impact endothelial function and vascular pathology. Disruptions in these mechanical forces can lead to plasma membrane breaches, impaired endothelial cell repair, and downstream effects like neointimal hyperplasia, which further distort flow and promote a pathological cycle.

The clinical consequences of these syndromes ultimately contribute to complications, such as venous stasis ulcers. This interdisciplinary review provides a synthesized framework for understanding how changes in biofluid mechanics lead to vascular remodeling and disease progression, supporting the development of improved diagnostic and therapeutic approaches.

## Introduction and background

Venous compression syndromes encompass a group of diseases in which veins are compressed by nearby anatomical structures, leading to impaired blood flow. These compressions may occur at the inguinal ligament, popliteal fossa, left renal vein, retrohepatic inferior vena cava, axillo-subclavian veins, internal jugular veins, common iliac veins, and external iliac veins [1]. These syndromes fall under the umbrella of chronic venous disease (CVD), which refers to a range of conditions in which veins are unable to efficiently return blood to the heart, most commonly from the lower limbs. Symptoms of CVD include leg pain, swelling, varicose veins, skin changes, and ulcers. CVD is classified using the CEP system (Clinical-Etiological-Anatomical-Pathophysiological), which stages the severity of disease from skin discoloration to active ulceration. Globally, CVD affects hundreds of millions of people, with over 30 million impacted in the United States alone, contributing to an economic burden of approximately $5 billion annually [2,3]. Clinically, it plays a role in around 200,000 cases of venous thrombosis and 180,000 amputations each year in the United States [4]. Enhancing our understanding of CVD could improve patient outcomes and alleviate its economic impact on the healthcare system.

Normal venous return relies on muscle contractions and one-way valves that prevent backflow. When external structures compress a vein, they can disrupt this system, elevate venous pressure, and impair drainage. One of the most recognized venous compression syndromes is May-Thurner Syndrome (MTS), classically defined as the compression of the left common iliac vein by the right common iliac artery against the lumbar spine [1,5]. This anatomical variant is relatively common, with up to 25% of the population experiencing >50% compression of the vein, though most remain asymptomatic [6]. However, among patients with lower-extremity deep vein thrombosis (DVT), approximately 2-3% are found to have MTS [6]. DVT, an acute condition involving clot formation in deep veins, differs from CVD but may be exacerbated by undiagnosed venous compression syndromes.

Diagnosing these syndromes can be difficult, as they lack reliable non-invasive markers. In MTS, for example, patients may present with recurring DVTs, yet physical exam, laboratory, and imaging findings are often nonspecific [5]. Invasive venography with intravascular ultrasound (IVUS) remains the gold standard [7], typically only pursued after other modalities fail. During venography, a catheter is inserted into a vein, and contrast dye is injected under fluoroscopy to outline the anatomical structure of the vessel. This provides a two-dimensional image of a relatively long segment of the vein, useful for identifying stenosis or flow abnormalities over a broader area. In contrast, IVUS involves inserting a catheter-mounted ultrasound probe that emits high-frequency sound waves to generate cross-sectional, three-dimensional images from within the vein. IVUS offers superior spatial resolution at the point of imaging, particularly for assessing vessel wall morphology and the degree of luminal narrowing. However, IVUS provides only localized information at the level of the transducer and is less widely available than venography. Used together, these modalities offer complementary anatomical perspectives that can enhance diagnostic accuracy. Overall, this diagnostic challenge is representative of venous compression syndromes as a whole in that many are only diagnosed after substantial pathology has already developed.

Although studies have explored the pathophysiology of venous compression and its impact on vascular biology, a comprehensive, interdisciplinary synthesis integrating biofluid mechanics, endothelial cell pathology, and molecular responses has not been performed. This scoping review aims to fill that gap by contextualizing venous compression syndromes through the lens of vascular physics, cell injury mechanics, and histopathology.

## Review

Biofluid mechanics

The dynamics of blood flow through veins are shaped by complex physical forces that differ from ideal fluid behavior. These forces are central to understanding venous compression syndromes, where altered flow conditions contribute to vascular dysfunction, endothelial injury, and remodeling. Fluid mechanics provides a framework to analyze how intravascular fluids respond to pressure gradients. A fluid element under normal force generates pressure in the x, y, and z directions, with small differences in pressure producing motion, deformation, or rotation [8]. Resistance to these movements is governed by viscosity, defined as a fluid’s internal resistance to shear deformation. Viscosity (η) is mathematically related to shear stress (τ, the tangential force per unit area) and shear rate (dv/dz, the velocity gradient perpendicular to flow) as per Equation 1:

$&tau;=&eta;&gamma;=&eta; (dv/dz)$ (1)

This linear model assumes Newtonian behavior, but blood is a non-Newtonian fluid due to its heterogeneous composition - plasma, cells, and proteins each with different velocities and densities. This complexity results in variable viscosity, which changes in response to local flow conditions, vessel geometry, and cellular distribution. Other forces influencing flow include tensional stress, compressional stress, lift forces, and drag forces [8,9]. The volumetric flow rate (Q), defined as the volume of blood moving through a cross-section per unit time, can be calculated as the product of cross-sectional area (A) and average velocity (v̄) as per Equation 2:

$Q = A &times; v̄$ (2)

This relationship is formalized by the Hagen-Poiseuille equation, which models the volumetric flow rate (Q) of an incompressible, Newtonian fluid through a rigid, cylindrical tube as shown in Equation 3 [8]. Here, ΔP is the pressure gradient along the vessel, r is the vessel radius, η is the dynamic viscosity, and L is the length of the tube. While this equation provides useful intuition for how changes in vessel radius or pressure gradient affect flow, its assumptions - laminar flow, constant viscosity, rigid geometry - are violated in most venous compression scenarios. In these settings, non-Newtonian flow behavior, turbulent patterns, and vessel compliance necessitate more complex modeling approaches. 

$Q = (&Delta;P&sdot;&pi;&sdot;r^4)/8&eta;L$ (3)

The Reynolds number predicts whether flow will be laminar or turbulent, while the Weissenberg number helps determine whether the fluid exhibits elastic (non-Newtonian) properties. In blood, especially under stress, high Weissenberg numbers and elevated Reynolds numbers often coexist, signaling a higher probability of turbulent flow patterns. In compressed venous segments, flow can shift from laminar to turbulent, causing shear stress gradients to become non-uniform. The relationship in Equation 1 assumes that shear rate decreases linearly with distance from the vessel wall and fails in this context. Instead, a curvilinear shear gradient emerges due to the heterogeneous distribution of cells. This phenomenon was shown in Phareus-Lindquist’s works, where it was appreciated that cells migrate to the vessel center, reducing viscosity near the wall and altering nutrient and gas exchange [10-12].

Flow disturbances are not limited to changes in viscosity and velocity. They often cause rotational motion of fluid elements, particularly in recirculation zones downstream of compressions. This rotational behavior can be measured macroscopically using circulation (Γ), which measures the tendency of fluids to rotate along a closed curve and is defined as the line integral of the tangential component of the velocity vector (V) over a path (ds) in Equation 4:

$&Gamma; = ∮ V &middot; ds$ (4)

At a microscopic scale, vorticity quantifies local rotation of fluid elements and is proportional to circulation per unit area. Both are altered in venous compression, especially downstream of the stenotic segment, where vortical flow dominates. Another important metric is the oscillatory shear index (OSI), which captures the proportion of the cardiac cycle during which wall shear stress reverses direction. Letting τw(t) denote instantaneous wall shear stress and T the cycle duration, OSI is given by Equation 5:

$OSI = 0.5 &times; [1 - (|&int;₀ᵀ &tau;w(t) dt| / &int;₀ᵀ |&tau;w(t)| dt)]$ (5)

While Equation 5 may seem overly detailed, the pertinent relation to vascular pathology is that a high OSI is associated with endothelial dysfunction, particularly increasing the risk of thrombus formation. 

Elevated viscosity from cell crowding increases peripheral resistance, raising upstream venous pressure. Over time, this disrupts oxygen delivery and nutrient exchange in the vessel wall and surrounding tissues. Initially, compensatory mechanisms may increase flow velocity to maintain perfusion. However, as per the Spencer model, beyond ~80% stenosis, this compensation fails - flow velocity declines and tissue ischemia ensues [10]. Table 1 summarizes the core fluid mechanical parameters discussed above and their clinical relevance to venous compression.

**Table 1:** Core Biophysical Concepts in Biofluid Mechanics

Term	Description	Clinical Implications
Hagen-Poiseuille Equation	Models laminar flow in rigid tubes	Not accurate for venous flow under compression
Reynolds Number	Determines the likelihood of turbulent vs laminar flow	Elevated in stenotic vessels or valvular dysfunction
Weissenberg Number	Predicts non-Newtonian fluid behavior under stress	High in blood under stress, indicating non-Newtonian behavior
Fahraeus-Lindqvist Effect	Erythrocytes concentrate at the vessel center	Alters shear at vessel wall in stenosed veins
Circulation (Γ)	Net rotation around a closed curve	Highlights macroscopic flow rotation
Oscillatory Shear Index (OSI)	Fraction of time shear stress is reversed	Associated with endothelial injury at higher values
Spencer Model	Describes relationship between stenosis and perfusion	Predicts drop in flow beyond ~80% compression

While such variations in viscosity and flow patterns are physiological under normal conditions, in venous compression syndromes, they become maladaptive. Disturbed flow may lead to local tissue hypoxia, inflammation, and endothelial injury - all precursors to pathological remodeling such as neointimal hyperplasia. 

Plasma membrane stressors

Endothelial cells that line blood vessels are uniquely positioned at the interface between circulating blood and vessel walls. These cells are constantly exposed to mechanical stressors, and their plasma membranes must maintain structural integrity while adapting to fluid dynamic forces. The frequency and intensity of these stressors determine whether the cell can recover, remodel, or progress to irreversible injury.

Biophysical Nature of the Plasma Membrane

The plasma membrane is primarily composed of phospholipids arranged in a bilayer, which forms a fluid, dynamic barrier with embedded proteins and cholesterol. The hydrophobic nature of phospholipids drives spontaneous aggregation in aqueous environments, promoting the formation of a three-dimensional, semi-permeable envelope [13,14]. Lipid molecules exhibit lateral and transmembrane fluidity, contributing to a heterogeneous and constantly shifting distribution of membrane domains.

This lipid heterogeneity is crucial for withstanding and responding to mechanical forces. Specific membrane microdomains, often enriched in cholesterol or sphingolipids (sometimes called lipid rafts), can have different viscosities and elastic properties, which in turn influence how they respond to deformation [15,16].

Types of Stressors

Stressors to the plasma membrane are broadly divided into chemical and biophysical. In venous compression syndromes, biophysical stressors are more relevant [15]. These include shear stress (r), which refers to tangential force from blood flow and varies by velocity gradients; compressional stress, which arises from external pressure on the vessel; stretch, which involves mechanical elongation or deformation of the membrane; and thermal stress, which occurs from acute temperature fluctuations. These forces act in a spatially heterogeneous manner across the membrane. For example, different domains of the endothelial membrane may experience varying degrees of shear stress, depending on vessel geometry, flow rate, and turbulence. This nonuniform stress distribution contributes to variable membrane viscosity and deformation responses across microdomains [16].

Cellular Responses to Membrane Injury

When a membrane is breached, the cell initiates a range of compensatory mechanisms [15-18]. The size and nature of the injury influence the repair response. Small breaches may spontaneously close via lipid rearrangement. For sub-100 μm injuries, cells may internalize the damaged region using caveolae-flask-shaped membrane invaginations enriched in caveolin proteins. This process removes the injured portion from the membrane surface without compromising cell integrity and is considered both a repair mechanism and a protective signaling response. For larger membrane injuries, the cell may fuse nearby intracellular vesicles to the damaged site; these vesicles coalesce at the lesion, forming a temporary seal. Cellular structures such as actin, annexins, or even organelle fragments may transiently plug the breach, especially in cases where permanent repair is delayed. Additional strategies, including exocytosis, contraction, constriction, and scission, are better characterized in other cell types, though endothelial cells may employ these under extreme stress. Each involves specific cytoskeletal or membrane-modifying pathways.

Thresholds of Cellular Injury

The outcome of plasma membrane stress is determined by its intensity and duration [12,13]. Low-frequency or low-magnitude stressors may result in reversible injury, with the potential for full recovery or adaptive remodeling. Remodeling includes changes in cell shape, cytoskeletal organization, or junctional protein distribution. In contrast, high-frequency or high-magnitude stressors, particularly those exceeding the membrane's repair capacity, lead to irreversible damage. This is often characterized by phospholipid peroxidation, breakdown of membrane integrity, increased permeability, and ultimately apoptosis or necrosis. These forms of injury are directly implicated in the early stages of endothelial dysfunction, which precedes more organized responses such as neointimal hyperplasia and thrombosis. Therefore, membrane stress is not merely a cellular event; it is a critical interface between hemodynamic forces and vascular pathology.

Endothelial cell pathology

Endothelial cells form the innermost lining of blood vessels and play a central role in vascular homeostasis. In venous compression syndromes, these cells are exposed to a spectrum of biomechanical stressors, including shear stress, compressional and rotational forces, and hydrostatic pressure, that disrupt their normal functions. These disturbances can lead to adaptive changes, reversible injury, or, in more severe cases, irreversible damage such as apoptosis or necrosis.

A key outcome of sustained endothelial stress is neointimal hyperplasia, which refers to the proliferation and migration of smooth muscle cells and fibroblasts into the tunica intima. This process is typically triggered by signals released in response to endothelial dysfunction and is exacerbated by aberrant hemodynamic forces. In compressed veins, the initial insult, such as disturbed flow or turbulent shear stress, can create focal endothelial injury. This damage, if not fully repaired, incites a reparative cascade involving inflammatory mediators, altered gene expression, and cell proliferation. The resulting hyperplastic tissue thickens the vessel wall and narrows the lumen, thereby further disrupting fluid dynamics [10,12].

One of the earliest changes in this pathophysiological cycle is the alteration of wall shear stress. Under conditions of laminar flow, shear stress promotes endothelial quiescence and alignment in the direction of flow. However, in areas of turbulent or oscillatory flow, common in venous compression syndromes, shear stress becomes spatially and temporally irregular. This promotes endothelial activation, pro-inflammatory signaling, and increased permeability. Over time, these changes can lead to focal breaches in the endothelial barrier, triggering a localized repair response that may evolve into neointimal hyperplasia.

This pathological remodeling is not merely structural but has functional consequences for blood flow. As the neointima encroaches on the vascular lumen, volumetric flow rate and shear rate are altered. Turbulent flow may become more pronounced, with greater velocity gradients and rotational stresses. These non-laminar patterns, including recirculation eddies and flow separation, are especially detrimental in venous segments with valvular incompetence or mechanical obstruction. The resulting biofluid environment favors a cycle of continued endothelial injury, inflammation, and remodeling. When this process occurs in veins, the clinical implications are significant. For instance, disruption of the hydrostatic pressure gradient impairs normal venous valve function, leading to venous hypertension. The pulsatile nature of venous return is diminished, and retrograde flow or reflux may develop. This is particularly relevant at venous valves, where endothelial cells are very sensitive to shear fluctuations and are disproportionately susceptible to injury from abnormal flow conditions [10,12,19].

Histopathological studies, primarily in animal models, have revealed distinctive features of endothelial remodeling under stress. Cells subjected to elevated shear stress often appear elongated, with disrupted intercellular junctions and altered cytoskeletal organization. Gene expression profiles also diverge depending on the type and magnitude of stress. For example, low shear stress upregulates pro-thrombotic and pro-inflammatory genes, while high shear stress tends to inhibit proliferation. Interestingly, endothelial cells from venous valves display a higher baseline expression of anticoagulant and anti-inflammatory molecules, yet this profile becomes dysregulated under disturbed flow. Furthermore, the transcriptional response of venous endothelial cells exposed to reflux or oscillatory flow shares similarities with that of arterial endothelial cells in atherosclerosis. These include increased expression of leukocyte adhesion molecules, matrix metalloproteinases, and mitogenic cytokines. These gene products promote inflammation, extracellular matrix degradation, and cellular proliferation - key drivers of chronic vascular pathology [12-14].

Endothelial cell pathology in venous compression syndromes represents the convergence of physical forces and biological responses. Neointimal hyperplasia, while initially a reparative attempt, ultimately compromises vessel integrity and perpetuates dysfunction. Understanding these cellular responses provides critical insight into the downstream sequelae of mechanical vein compression, including thrombosis, valve failure, and venous stasis disease.

Interdisciplinary discussion 

Venous compression syndromes present a complex pathophysiological scenario that requires an interdisciplinary understanding of biofluid mechanics, endothelial biology, and clinical vascular pathology. At the core of these syndromes lies the deviation of venous blood flow from idealized conditions. Blood, a non-Newtonian fluid composed of plasma, cells, proteins, and lipids, demonstrates variable viscosity under stress. This viscosity is influenced by shear rate, cellular concentration, and vessel geometry, which in turn affect the distribution of hemodynamic forces across the endothelium. Turbulent and non-laminar flow, common in areas of venous compression, further complicates this hemodynamic environment. Flow disturbances increase both shear stress gradients and rotational forces, quantified macroscopically as circulation and microscopically as vorticity, which are transmitted directly to the endothelial surface. These forces act as biophysical stressors, capable of deforming the plasma membrane and initiating a cascade of cellular responses. Depending on the specific membrane microdomain affected and the magnitude of applied stress, phospholipids may resist deformation or yield to form a plasma membrane breach.

When such a breach occurs, endothelial cells attempt to restore membrane integrity through a variety of mechanisms. For small injuries under 100 nm, caveolar endocytosis may be used to internalize and remove damaged portions of the membrane. Larger breaches require patching, where intracellular vesicles fuse at the lesion site, or plugging, a temporary solution involving cytoskeletal or vesicular elements. While these mechanisms offer temporary relief, repeated or severe insults overwhelm the repair capacity, resulting in either reversible remodeling or irreversible damage leading to apoptosis or necrosis. Reversible injury may lead to cytoskeletal reorganization, elongation of endothelial cells, and discontinuity in junctional proteins. These changes are associated with altered gene expression, including upregulation of adhesion molecules, inflammatory cytokines, and proteases. In contrast, irreversible injury disrupts membrane permeability, triggers oxidative stress, and compromises cell survival. These pathological shifts in endothelial behavior are amplified by the underlying flow disturbances.

The downstream consequence of these changes is often neointimal hyperplasia, which narrows the venous lumen and worsens hemodynamic disruption. Abnormal flow patterns such as eddies, reflux, and flow separation, often associated with an elevated OSI, intensify this remodeling, particularly in areas of incompetent valves or chronic obstruction. As the neointima thickens, shear stress is further distorted, creating a vicious cycle of injury and remodeling. This phenomenon can be contextualized using the equation of continuity, which describes the conservation of mass in fluid dynamics: as vessel diameter narrows, fluid velocity must increase to maintain constant flow. In areas of venous compression, this increase in velocity occurs alongside changes in pressure and wall shear stress, contributing to focal endothelial injury and downstream remodeling. This cycle has several clinical implications. Chronic venous compression elevates intraluminal pressure, reduces venous diameter, and increases vascular resistance. The resulting venous hypertension interferes with normal valve closure, impairs pulsatile venous return, and promotes stasis. These conditions contribute to pain and swelling, as well as thrombotic risk and tissue hypoxia. The transcriptional response of endothelial cells under such conditions resembles that seen in atherosclerotic arteries. Both environments feature enhanced leukocyte recruitment, increased extracellular matrix turnover, and sustained inflammatory signaling. These shared features underscore the pathological potency of disturbed flow and the central role of mechanical forces in chronic vascular disease. While DVT and venous compression syndromes are pathophysiologically distinct, there is an overlap in the endothelial responses observed. Both conditions can result in localized inflammation, endothelial denudation, and upregulation of prothrombotic mediators. However, compression-related injury is more often characterized by chronic remodeling, intimal thickening, and venous wall fibrosis, whereas DVT typically presents with acute thrombosis and luminal obstruction.

**Figure 1:**
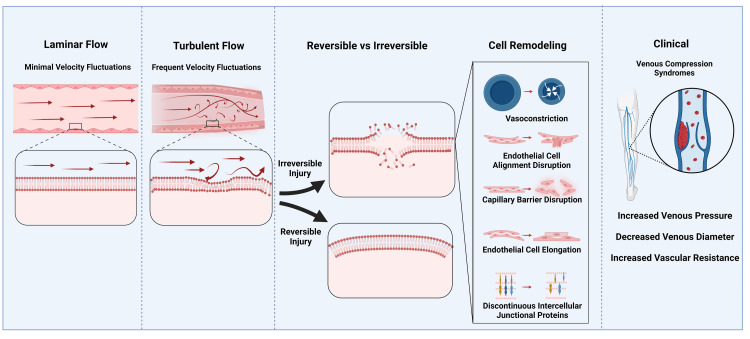
Biophysical and Pathological Framework of Venous Compression Syndromes. This figure is the original work of the authors.

The cumulative effect of ongoing endothelial injury, tissue remodeling, and impaired hemodynamics may lead to the formation of intravenous spurs, progressive venous stenosis, and the development of collateral veins. Additionally, extravasation of intravascular fluid into the interstitium can provoke venous stasis dermatitis, where breakdown of erythrocytes by macrophages contributes to hemosiderin deposition and local inflammation. In advanced cases, these processes culminate in venous stasis ulcers. There are similar findings in MTS from a histopathologic perspective, including intimal fibrosis, elastin fragmentation, and focal endothelial disruption at the site of compression. Figure 1 provides a schematic overview of the pathophysiologic progression of venous compression syndromes, from abnormal biofluid mechanics to endothelial injury, and clinical sequelae.

Limitations

While this review presents an integrative framework for understanding the pathophysiology of venous compression syndromes, several limitations should be noted. Some of the described mechanisms, particularly those involving endothelial remodeling, are derived from animal studies or highly controlled experimental models. As such, caution is warranted when extrapolating these findings to human physiology. Moreover, this manuscript was conceived as an interdisciplinary scoping review rather than a formal systematic review, due to the broad and mechanistically diverse nature of the topic spanning vascular biology, fluid dynamics, and clinical syndromes. As such, a rigid PRISMA-style framework was not applied. Instead, a targeted, iterative search strategy was employed using PubMed. Key terms included combinations of "venous compression," "May-Thurner syndrome," "chronic venous disease," "shear stress," "neointimal hyperplasia," and "endothelial injury." References were included based on their relevance to central themes of venous flow disruption and vascular remodeling. While this limits replicability by systematic review standards, we believe this approach is appropriate given the conceptual and interdisciplinary aims of the manuscript. These limitations are consistent with the narrative scope of this work but should inform the interpretation of its conclusions.

This multifaceted interplay between mechanical, cellular, and pathological factors illustrates why venous compression syndromes demand an integrative approach. Understanding how physical forces initiate and propagate vascular disease enables more precise diagnostic strategies and may inform therapeutic targets that address not only anatomic obstruction but also the cellular consequences of altered flow.

## Conclusions

Venous compression syndromes are important yet underdiagnosed contributors to CVD. This review integrates concepts from vascular biology, hemodynamics, and clinical medicine to describe how abnormal flow patterns can injure the endothelium, disrupt membrane integrity, and promote maladaptive remodeling. A central pathological feature is neointimal hyperplasia, which contributes to progressive venous narrowing, valvular dysfunction, and venous hypertension. Understanding this process has practical implications. Improved diagnostic tools, such as earlier use of IVUS, may identify compression earlier. Therapeutically, targeting endothelial dysfunction and limiting hyperplasia may help interrupt the cycle of mechanical injury and vascular remodeling.

These insights rely on contributions from multiple disciplines. Fluid mechanics offers models for predicting flow disruption; vascular biology explains endothelial responses; and clinical medicine contextualizes these changes in patient outcomes. Collaboration across these domains is essential for advancing diagnostics, refining interventions, and developing predictive models of disease progression. This work is limited by some studies using animal models and in vitro data, and by the absence of a systematic literature review. Future research should focus on translational studies in human venous tissue under physiologic and pathologic flow conditions. Specific unanswered questions include identifying early biomarkers of endothelial stress, defining flow thresholds for irreversible damage, and understanding patient-specific risk factors. Addressing these gaps could improve patient care and reduce the considerable healthcare costs associated with delayed recognition and treatment of venous compression syndromes.
